# Propolis mitigates histopathological alterations in the pituitary gland and reproductive system of female albino rats subjected to cadmium toxicity

**DOI:** 10.14202/vetworld.2025.1466-1478

**Published:** 2025-06-10

**Authors:** Abdulla A. Albishtue, Aqeel Mohsin Al-Mahmmodi, Hasan A. Almamoori, Mustafa Ali Alahmer

**Affiliations:** 1 Department of Anatomy and Histology, Faculty of Veterinary Medicine, University of Kufa, Najaf, Iraq; 2 Department of Clinical Sciences, Faculty of Veterinary Medicine, University of Kufa, Najaf, Iraq

**Keywords:** antioxidant enzymes, estradiol, ovarian histology, pituitary gland, propolis, cadmium chloride, reproductive toxicity

## Abstract

**Background and Aim:**

Cadmium (Cd) is a pervasive environmental toxin that disrupts endocrine function and induces oxidative damage in reproductive organs. Propolis (PRO), a resinous substance produced by bees, has garnered attention for its antioxidant and estrogenic properties. This study investigated the protective potential of PRO on the pituitary-ovarian-uterine axis in female rats subjected to Cd-induced toxicity.

**Materials and Methods:**

Thirty adult female albino rats were randomized into five groups (n = 6/group): Control (C), Cd-only (T0), and Cd plus PRO at 150, 300, and 500 mg/kg body weight (BW) (T1–T3, respectively). Cadmium chloride was administered orally at 5 mg/kg for 4 weeks. PRO was co-administered daily through gavage. At the proestrus stage, animals were euthanized for tissue collection. Vaginal cytology was used to confirm estrous stage. Histopathological examination of the ovary, uterus, and pituitary gland was performed using H&E staining. Serum estradiol (E2) and superoxide dismutase (SOD) activity were assessed to evaluate hormonal and oxidative responses. Morphometric measurements were statistically analyzed through one-way analysis of variance with Tukey’s *post hoc* test.

**Results:**

Cd exposure (T0) led to prolonged estrous cycles, ovarian atresia, uterine degeneration, and significant disruption of pituitary architecture, accompanied by reduced E2 and SOD levels (p < 0.05). PRO administration dose-dependently ameliorated these alterations. The highest PRO dose (T3) restored the histological architecture of all target organs to near-normal levels, significantly increased ovarian and uterine weight ratios, and elevated both E2 and SOD activity. Histomorphometric analysis confirmed increased follicle survival, thickened ovarian surface epithelium, and elevated interstitial cell counts. Pituitary endocrine cell counts and uterine gland numbers were also significantly higher in PRO-treated groups, particularly T3.

**Conclusion:**

PRO supplementation at 500 mg/kg BW significantly attenuates Cd-induced reproductive and endocrine toxicity in female rats by restoring histological integrity and enhancing antioxidant and estrogenic responses. These findings suggest PRO as a promising candidate for mitigating heavy metal-induced reproductive dysfunction.

## INTRODUCTION

Heavy metal contamination of the environment poses a significant threat to ecosystems and public health, exerting toxic effects on both humans and animals, and potentially resulting in fatal outcomes. In response to this global concern, increasing research attention has been directed toward developing novel therapeutic strategies to mitigate the toxic effects of heavy metals [1]. Among these, cadmium (Cd) is considered one of the most hazardous due to its widespread release into the environment from both anthropogenic and natural activities, including the refining and smelting of non-ferrous metals, waste management, the manufacture of fertilizers, plastics, batteries, and pigments [2, 3]. Human exposure to Cd primarily occurs through tobacco smoke, inhalation of industrial emissions, and the ingestion of contaminated food, such as seafood [4, 5]. Its high environmental persistence and non-biodegradable nature facilitate bioaccumulation, allowing it to move efficiently through the food chain [6].

Once introduced into the body through the gastrointestinal or respiratory tract, Cd enters systemic circulation and preferentially accumulates in critical organs, including the lungs, kidneys, pancreas, testes, and ovaries [7]. Extensive studies have highlighted the endocrine-disrupting potential of Cd, classifying it as an endocrine-disrupting chemical (EDC). These substances, often referred to as exogenous compounds, interfere with normal hormonal regulation and can adversely affect both the exposed organism and its progeny [8, 9].

Cd toxicity is primarily mediated through mitoch-ondrial dysfunction and oxidative stress. The body’s inability to effectively excrete Cd results in prolonged biological retention, which disrupts mitochondrial ATP production and increases reactive oxygen species (ROS) levels. This oxidative insult leads to structural and functional damage in ovarian and uterine tissues, which are particularly vulnerable [10, 11]. Elevated ROS also impair macromolecules such as DNA and proteins, disturb the antioxidant defense system, and alter mitochondrial homeostasis, autophagy pathways, and epigenetic regulation [12].

The ovaries, due to their high metabolic rate and energy demands, are particularly susceptible to Cd-induced cytotoxicity. Zenzes *et al*. [13] reported Cd concentrations reaching 6.73 ± 0.31 μg/L in the follicular fluid of women, which correlated with increased follicular atresia, impaired folliculogenesis, failed implantation, spontaneous abortion, and ovula-tory dysfunction [8, 14]. As reviewed by Thompson and Bannigan [15], Cd exposure detrimentally affects gametogenesis in both sexes, contributing to impl-antation failure and embryonic lethality. In addition, rodent studies by Blum *et al*. [16] and Blum *et al*. [17] have demonstrated the capacity of Cd nanoparticles to traverse the placental barrier, thereby disrupting fetal development.

In recent decades, considerable interest has been directed toward exploring the therapeutic roles of natural products in mitigating heavy metal toxicity. Propolis (PRO), a resinous substance collected by bees from plant exudates, has emerged as a candidate of particular interest due to its complex bioactive profile and traditional use as a health remedy [18]. Contemporary research has substantiated many of these traditional claims, demonstrating that PRO exerts protective effects against Cd-induced hepatorenal and reproductive toxicity in animal models [19, 20]. Its efficacy is largely attributed to its antioxidant, regenerative, and endocrine-regulatory properties [21, 22]. Notably, bioactive constituents such as artepillin C, chrysin, and caffeic acid phenethyl ester contribute to its cytoprotective functions, including demonstrated anti-cancer activity in cell lines derived from gastrointestinal, respiratory, and reproductive tissues [23].

Although the toxicological impacts of Cd on the reproductive and endocrine systems are well-documented, particularly its capacity to induce oxidative stress, hormonal dysregulation, and histopathological alterations, current research remains limited regarding the role of natural antioxidants in counteracting these effects within the entire pituitary-ovarian-uterine axis. While several studies have evaluated the protective effects of natural compounds such as vitamins, quercetin, and flavonoids on isolated reproductive tissues, comprehensive investigations addressing the histomorphological and functional restoration of the reproductive axis under Cd-induced toxicity are scarce. Furthermore, despite growing evidence supporting the therapeutic potential of PRO due to its antioxidant and phytoestrogenic properties, few studies have systematically examined its dose-dependent efficacy in restoring hormonal balance, redox homeostasis, and histoarchitecture across the pituitary gland, ovaries, and uterus. The lack of integrated assessments that include hormonal profiling, antioxidant status, and tissue-specific morphometry under controlled experimental conditions constitutes a critical knowledge gap in toxicological research and therapeutic development.

This study aimed to evaluate the protective efficacy of PRO against Cd chloride-induced toxicity in the pituitary-ovarian-uterine axis of adult female rats. Specifically, the study assessed the histomorphological changes in the ovary, uterus, and pituitary gland, quantified serum estradiol (E2) levels and superoxide dismutase (SOD) activity, and analyzed the dose-dependent ameliorative potential of PRO administered at 150, 300, and 500 mg/kg body weight (BW). By integrating hormonal, oxidative, and histological endpoints, this research provides mechanistic insights into the therapeutic role of PRO in mitigating heavy metal-induced reproductive and endocrine dysfunction.

## MATERIALS AND METHODS

### Ethical approval

All experimental procedures involving animals were conducted in accordance with institutional guidelines for the care and use of laboratory animals and approved by the Institutional Animal Care and Use Committee of the University of Kufa (Approval No.: University of Kufa/IACUC/AUPR13775/2024).

### Study period and location

Thirty adult female albino rats were administered once daily with Cd and PRO for a period of 4 weeks (1 June 2024 to 28 June 2024). The study was conducted at the Faculty of Veterinary Medicine, University of Kufa.

### Preparation of PRO extract

Commercially sourced PRO was prepared as per the method described by Hendi *et al*. [24]. Briefly, 10 g of raw PRO was soaked in 100 mL of distilled water in a dark brown glass container and kept at room temperature (20°C) for 24 h in the dark. The solution was shaken every 2–3 h daily for 2 weeks using a magnetic stirrer placed on a hot plate maintained at 45°C. After the extraction period, the solution was allowed to cool to 20°C before administration. The final dosage of PRO administered to rats was adjusted based on BW.

### Preparation of cadmium chloride (CdCl_2_) solution

CdCl_2_ (molecular weight 183.32 g/mol) was obtained from R&K Chemicals, UK. A working solution was prepared in distilled water and administered orally to rats at a dose of 5 mg/kg BW via gavage for four consecutive weeks [10].

### Experimental design

Thirty adult female albino rats (12 weeks old) were obtained from the Animal Resources Center, Faculty of Veterinary Medicine, University of Kufa. Animals were acclimatized for 14 days under standard laboratory conditions (22°C ± 2°C, 55% ± 10% humidity, 12-h light/dark cycle) with ad libitum access to feed and water.

Rats were randomly divided into five groups (n = 6 per group):

- Control group (C): Received distilled water.
- T0: Received CdCl_2_ (5 mg/kg BW) without PRO.
- T1: Received CdCl_2_ + PRO (150 mg/kg BW).
- T2: Received CdCl_2_ + PRO (300 mg/kg BW).
- T3: Received CdCl_2_ + PRO (500 mg/kg BW).

Cd and PRO were co-administered orally for 28 consecutive days. The selected PRO dosages were based on previous reports by Teles *et al*. [25], Salehi *et al*. [26], and Sheir *et al*. [27], and the Cd dose followed the protocol of Quddus *et al*. [11]. At the end of the experimental period, all animals were euthanized at the proestrus stage using CO_2_ asphyxiation followed by anesthesia with ketamine (30 mg/kg BW) and xylazine (10 mg/kg BW) to facilitate blood and tissue collection [28].

### Estrous cycle synchronization and vaginal cytology

To synchronize estrous cycles, each rat received two intramuscular injections of Estrumate, spaced 3 days apart, before the start of the experiment. Vaginal smears were collected daily for 4 weeks and evaluated microscopically to monitor estrous cyclicity. Cytological evaluation was performed using image analyzer software as described by Albishtue *et al*. [29].

### Histomorphological analysis

Post-euthanasia, the pituitary gland, ovaries, and uterus were dissected, weighed, and subjected to gross examination. Tissues were fixed in 10% neutral-buffered formalin for 24 h and processed for hematoxylin and eosin (H&E) staining. Ovarian morphometric analysis was performed following the method of Albishtue *et al*. [30] with minor modifications. Serial sections from the central to peripheral regions were prepared for microscopic analysis.

The number and classification of ovarian follicles (primordial, primary, secondary, and antral) were determined based on oocyte morphology and the number of granulosa cell layers. Atretic follicles were identified by oocyte degeneration and granulosa disorganization [30]. The thickness of the ovarian surface epithelium (OSE) and the number of interstitial cells were quantified using digital image analysis.

For uterine samples, histological changes in the luminal epithelium (LE), glandular epithelium (GE), and endothelial lining were evaluated microsco-pically [29, 31]. The number of uterine glands and the thickness of LE and GE were measured using an Olympus image analyzer. Pituitary endocrine cells in the pars distalis were quantified at 40× magnification, while epithelial thicknesses were assessed at 100× magnification. Three random measurements per structure per animal were recorded [29–31].

### Serum hormone assays

At the proestrus phase, 1 mL of blood was collected through cardiac puncture from each rat into EDTA-coated tubes. Serum was separated by centrifugation at 699× *g* for 10 min at 4°C and stored at −20°C until analysis. E2 concentrations were determined using a competitive immunoluminometric assay (Maglumi Estradiol Kit, Maglumi X3, China) following the manufacturer’s protocol.

### Assessment of oxidative stress markers

Serum SOD activity was assessed as a biomarker of oxidative stress using the Enzychrom™ SOD Assay Kit (Solarbio, China). The assay quantifies the dismutation rate of superoxide radicals, reflecting SOD enzymatic activity [32].

### Statistical analysis

Data were expressed as mean ± standard error of the mean. Statistical comparisons among groups were performed using one-way analysis of variance followed by Tukey’s *post hoc* test. Analyses were conducted using GraphPad Prism version 6.0 (GraphPad Software, San Diego, CA). p < 0.05 was considered statistically significant.

## RESULTS

### Effects of PRO treatment on vaginal cytology and reproductive organ weights

Vaginal cytological evaluation revealed three predominant cell types: Nucleated epithelial cells, leukocytes, and cornified squamous epithelial cells. Based on the relative abundance of these cells, the stage of the estrous cycle was identified. At proestrus, smears predominantly displayed rounded nucleated epithelial cells with oval nuclei and lightly stained cytoplasm. The control and PRO-treated groups maintained a regular estrous cycle of approximately 4 days.

**Figure 1:**
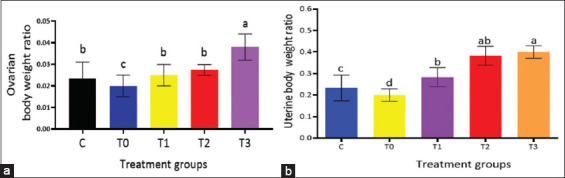
Impact of propolis on (a) the ovarian body weight ratio and (b) uterine body weight ratio in rats subjected to cadmium toxicity. Notice that T3 had the highest ratio values among all experimental groups. Means ± standard errors is used to express the data. Significant differences at p < 0.05 are indicated by different letters (a, b, c and d).

Conversely, rats in the Cd-only group (T0) exhibited prolonged and irregular estrous cycles lasting 6–7 days, although no qualitative alterations in smear morphology were detected. By day 28, significant diff-erences were observed in both the ovarian and uterine BW ratios among experimental groups. The T3 group (500 mg/kg BW PRO) showed the highest ovarian and uterine BW ratios (p < 0.05), while the T0 group had the lowest (Figure 1).

### Effects of PRO on ovarian histoarchitecture

**Figure 2:**
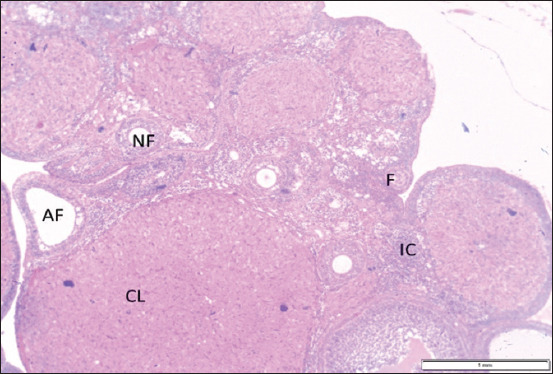
Histological sample of ovarian structure are obvious different ovarian follicles, Cl, interstitial cells, and ovarian surface epithelium (H and E stain, × 10). NF=Antral follicle, AF=Atretic follicle, F=Follicular unit, IC=Interstitial cell, CL=Corpus luteum.

**Figure 3:**
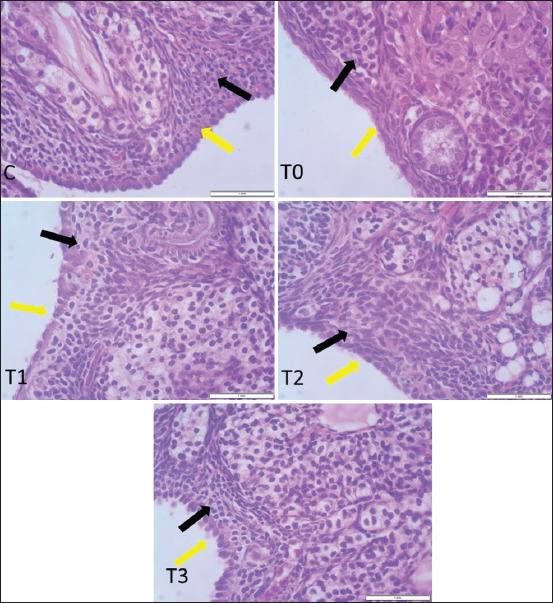
Impact of propolis supplementation on ovarian histomorphology in rats exposed to cadmium toxicity. Notice, different types of ovarian surface epithelium (yellow arrow) and interstitial cells (black arrow) in all groups (H&E stain, 40× magnification).

Gross examination of ovarian samples revealed no visible pathological alterations in any group. Microscopic evaluation, however, demonstrated distinct histological differences. In the control group (C), the OSE was simple cuboidal, with centrally placed spherical nuclei and eosinophilic cytoplasm. In T0, the OSE appeared flattened and squamous, indicative of degenerative changes. The epithelium in T1 and T2 was similar to the control, while in T3, it transitioned to a simple columnar morphology with elongated heterochromatic nuclei (Figures 2 and 3).

**Figure 4:**
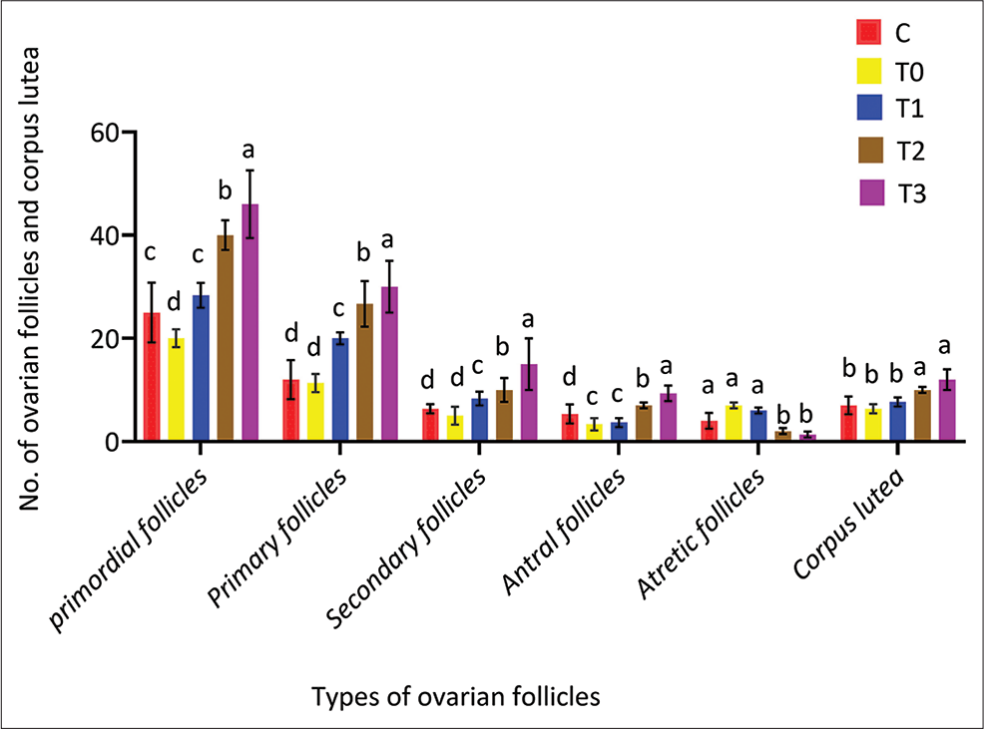
Impact of propolis supplementation on follicular development in rats exposed to cadmium toxicity. Rats were scarified at the pro-estrus stage. Error bars with different alphabets (a, b, c, and d) indicate statistically significant differences (p < 0.05) for all types of ovarian follicles and corpus lutea.

Histomorphometric analysis of follicular development showed significantly higher counts of primordial, primary, secondary, and antral follicles in the T3 group compared to other groups (p < 0.05), alongside a greater number of corpora lutea (Figure 4). In contrast, T0 demonstrated the highest number of atretic follicles. PRO administration improved follicular survival in a dose-dependent manner.

As shown in Table 1, T3 also exhibited the greatest OSE thickness and the highest number of interstitial cells (p < 0.05). These parameters were significantly elevated compared to T0 and other treatment groups, suggesting enhanced ovarian regeneration.

**Table 1:** Impact of PRO on histomorphometric parameters of the ovary during the proestrus stage of the estrous cycle in rats exposed to cadmium.

Parameters	C	T0	T1	T2	T3
Thickness of the OSE (un)	7.13 ± 0.34^b^	3.75 ± 0.50^c^	6.80 ± 0.30^d^	7.47 ± 0.32^b^	10.33 ± 0.36^a^
Number of interstitial cells	120.00 ± 3.33^b^	75.50 ± 2.66^d^	95.67 ± 3.22^c^	118.17 ± 4.34^b^	150.00 ± 2.45^a^

All rat uterine values were lower (p < 0.05) in the PRO-supplemented group T0 and higher in PRO-supplemented T3. Means ± standard errors are used to express data. a, b, c, and d within rows indicate significant differences at p < 0.05. OSE=Ovarian surface epithelium, un=micrometer, PRO=Propolis. (40× magnification).

### Histological effects of PRO on the pituitary gland

**Figure 5:**
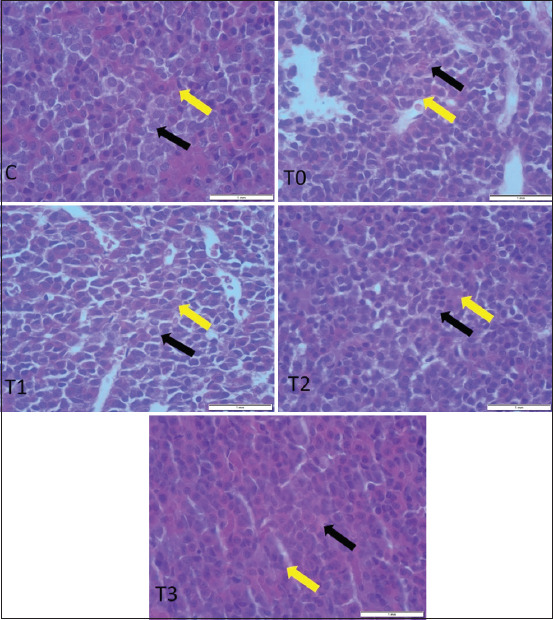
Impact of propolis on histomorphological samples collected from rat pituitary glands exposed to cadmium. Degenerative alterations and cell cord disorganization were observed at T0. However, groups T1 and T2 showed reduced degenerative changes. The T3 group appeared like C. The components of the pars distalis are active endocrine cells (yellow arrow) and blood vessels (black arrow) (H&E; 40× magnification).

Representative histological sections of the pituitary gland are depicted in Figure 5. In the control group, the pars distalis exhibited regular polygonal endocrine cells with clear cytoplasm and rounded nuclei. In contrast, T0 showed marked degenerative changes, including edema, disrupted cell cords, hypochromic cytoplasm, and pyknotic nuclei.

Dose-dependent attenuation of histopathological lesions was observed in the PRO-treated groups, with T3 displaying pituitary architecture comparable to the control. Furthermore, the T3 group exhibited a significantly higher number of active endocrine cells in the pars distalis relative to all other groups (p < 0.05, Table 2), indicating improved pituitary function.

**Table 2:** Impact of PRO on histomorphometric parameters of pituitary glands during the proestrus stage in rats exposed to cadmium.

Parameters	C	T0	T1	T2	T3
No. of endocrine cells	90.00 ± 4.32^b^	50.40 ± 2.74^d^	62.33 ± 6.09^c^	85.22 ± 7.50^b^	113.67 ± 4.76^a^

The number of endocrine cells in the pituitary glands was lower (p < 0.05) in T0 and higher in PRO-supplemented T3. Means ± standard error of the mean are used to express data. a, b, c, and d within rows indicate a significant difference at p *<* 0.05. (40× magnification), PRO=Propolis

### Histological impact of PRO on uterine architecture

**Figure 6:**
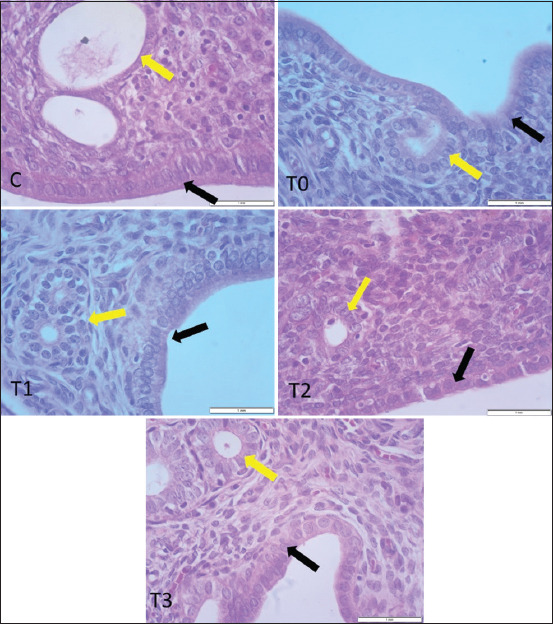
Histological samples from uteri exposed to cadmium chloride toxicity in adult rat. Notice destruction of uterine luminal epithelium and uterine gland necrosis in positive controls T0, T1, and T2. The uterine glands are indicated by yellow arrows, whereas the lining of the uterus is indicated by black arrows (H&E;40× magnification).

Macroscopic evaluation revealed no gross abnormalities in uterine tissues across all groups. However, histological analysis demonstrated inflam-matory lesions, including endometrial atrophy, vacuolar degeneration of luminal epithelial cells, and reduced glandular integrity in T0, T1, and T2. In contrast, T3 and control animals exhibited well-preserved uterine architecture, including intact luminal and GE (Figure 6).

Quantitative analysis revealed significantly grea-ter uterine gland numbers and increased epithelial thickness in T3 compared to T0 and intermediate-dose groups (p < 0.05, Table 3).

**Table 3:** Impact of PRO on histomorphometric parameters of uterine structures during the the proestrous stage in rats exposed to cadmium.

Parameter	C	T0	T1	T2	T3
Thickness of GE (µm)	13.23 ± 2.59^c^	9.50 ± 0.84^d^	13.67 ± 1.36^c^	21.52 ± 2.62^b^	26.82 ± 1.33^a^
Thickness of LE (µm)	25.00 ± 1.93^ab^	13.67 ± 0.49^d^	21.00 ± 1.93^c^	28.00 ± 1.32^b^	31.83 ± 1.20^a^
Thickness of Endothelium (µm)	661.35 ± 20.86^c^	422.33 ± 10.20^d^	653.50 ± 33.59^c^	720.50 ± 30.10^b^	785.54 ± 11.45^a^
No. of uterine glands	42.33 ± 2.35^a^	22.05 ± 3.20^c^	27.67 ± 3.68^b^	30.67 ± 4.40^b^	41.20 ± 2.00^a^

All rat uterine values were lower (p < 0.05) in T0 and higher at T3. Means ± standard errors are used to express data. a, b, c, and d within rows indicate significant differences at p *<* 0.05. LE=Uterine luminal epithelium, GE=Uterine glandular epithelium, PRO=Propolis

### Effects of PRO on E2 concentration

**Figure 7:**
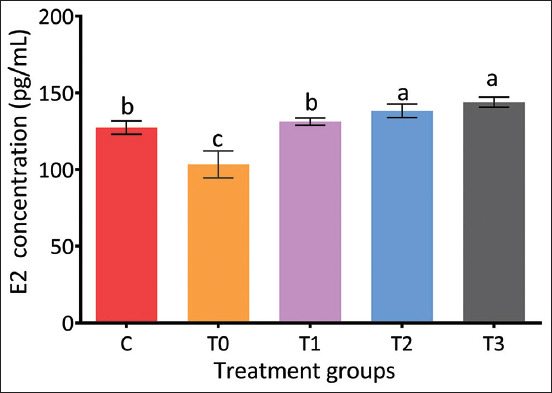
Impact of propolis on E2 hormone levels in rats exposed to cadmium chloride. The E2 concentration was highest in T3 (p < 0.05). On the other hand, T0 had the notably lowest E2 concentration. Means ± standard errors are used to express data.

As illustrated in Figure 7, PRO supplementation significantly increased E (E2) concentrations in a dose-dependent manner. The T3 group exhibited the highest E2 levels (p < 0.05), while T0 recorded the lowest. Among the intermediate doses, T2 demonstrated the greatest improvement, although still lower than T3. These findings suggest enhanced steroidogenic activity with PRO treatment.

### Effects of PRO on SOD activity

**Figure 8:**
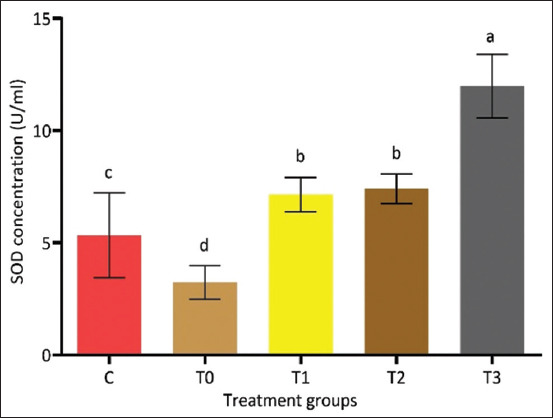
Impact of PRO on superoxide dismutase activity in rat serum after exposure to cadmium chloride. SOD concentrations were higher in T3 and lower (p < 0.05) in the PRO unsupplemented group T0. Means ± standard errors are used to express data. A significant difference at p < 0.05 is indicated by different letters for a and b within rows. Note: SOD=Superoxide dismutase, PRO=Propolis.

Serum SOD activity was significantly reduced in the T0 group relative to the control. However, PRO supplementation markedly restored SOD activity in a dose-dependent manner (Figure 8). The T3 group demonstrated the highest enzymatic activity (p < 0.05), suggesting potent antioxidant defense enhancem-ent and redox stabilization through PRO-mediated intervention.

## DISCUSSION

### Cd-induced endocrine disruption and estrous cycle irregularities

Changes in the cellular composition of the vaginal mucosa reflect underlying endocrine events. Cd functions as an endocrine disruptor, adversely affecting reproductive physiology and contributing to fertility disorders. This study revealed that rats exposed to Cd displayed prolonged and irregular estrous cycles. This result corresponds to the findings of an earlier study by da Costa *et al*. [33], which found a strong positive correlation between serum and reproductive tract (ovary and uterus) Cd levels and the length of the estrous cycle, atretic follicles, and inflammation of the reproductive tract. However, histological observations from this study indicated that the PRO-treated and control groups maintained a normal estrous cycle, suggesting a regulatory effect on reproductive cyclicity. On the other hand, Nasiadek *et al*. [34] revealed that Cd causes histopathological alterations in the female reproductive system associated with disruptions of steroid hormone synthesis and the estrous cycle according to a prior study, Cd may have a negative impact on uterine physiological processes by harming the uterine glands, which can change endometrial glandular secretions such as hormones, transport proteins, growth factors, cytokines, and enzymes necessary for conceptus development [35].

### Molecular pathways modulated by PRO

Specific molecular mechanisms such as estrogen receptor (ER) modulation, VEGF-mediated proliferation, caspase-3 inhibition, and SOD activation were incorporated to explain how PRO counteracts Cd toxicity. Menstrual irregularities, such as amenorrhea and oligomenorrhea, are frequently associated with decreased estrogen levels [36]. According to recent research, Cd binds to ERs. As a result, this metal is referred to as metalloestrogen [37]. Oral PRO administration has been shown to induce estrogenic activity in organs of the body that express ERs, suggesting that it may be a useful treatment for menopausal symptoms [38]. Selective ER modulators (SERMs) are a broad class of non-steroidal substances that act as ligands for ERs. However, SERMs have the rare capacity to selectively act as agonists or antagonists in a target gene and in a tissue-specific manner, in contrast to estrogens that work as ER agonists with essentially different potencies [39, 40]. Therefore, the mixed agonism/antagonism profile of SERMs prov-ides advantageous estrogenic actions in target tissues while avoiding negative, off-target effects, which is their pharmacological advantage. SERMs are clinically used to prevent osteoporosis and to preserve healthy serum lipid profiles in postmenopausal women [41]. Flavonoids present in PRO exhibit structural similarity to selective ER modulators, enabling them to exert tissue-specific estrogenic activity [42].

### Apoptosis regulation and VEGF signaling

Numerous essential cellular functions, such as apoptosis, proliferation, differentiation, and the inflammatory response, are mediated by the evolu-tionarily conserved family of cysteine-dependent prote-ases known as caspases. Numerous diseases, including inflammatory diseases, neurological disorders, metab-olic diseases, and cancer, have been related to the pathophysiology of the dysregulation of caspase-mediated apoptosis and inflammation [43]. CdCl_2_ exposure upregulated caspase-3 expression, whereas PRO supplementation significantly downregulated its expression [44]. Vascular Endothelial Growth Factor (VEGF) is responsible for the angiogenesis process in ovarian and uterine structures, such as the follicle, stromal cells, corpus luteum, and uterine glands. The-refore, VEGF is a crucial factor in the development, maintenance, and degradation of these structures and intensifying the vascularization of uterine glands, the follicle, stromal cells, and the corpus luteum, allowing the entrance of nutrients [45, 46]. A previous study by Christenson and Stouffer [47] reported that granulosa cells directly promote VEGF synthesis in response to follicle-stimulating hormone (FSH)-like and luteinizing hormone (LH)-like gonadotropins. Previous studies by Zarei *et al*. [48] and Ernawati and Puspasari [49] revealed that PRO can induce and increase the expression of VEGF. VEGF, FSH, and estradiol synergistically inhibit caspase-3 activation, promoting cell survival and proliferation [50]. Therefore, PRO is a food additive that lessens the tissue damage caused by CdCl_2_.

### Oxidative stress and SOD activation by PRO

Previous studies have reported that Cd causes damage to the ovarian follicles and uterus. One possible explanation for this could be the disruption of reproductive hormone synthesis and an increase in ROS [11, 51]. SOD, an essential endogenous antioxidant enzyme that acts as the body’s first line of defense against ROS by scavenging free radicals and averting oxidative tissue damage, is the most significant and effective detoxification enzyme in cells [52, 53]. Exposure to Cd has been shown to increase lipid peroxidation and inhibit SOD functioning, which can lead to oxidative damage in the body’s organs [54]. According to Nasiadek *et al*. [55], one of the primary ways that heavy metal toxicants, such as Cd, harm the reproductive system is by disrupting the body’s equilibrium between antioxidants and ROS, which results in oxidative stress. This study demonstrated that rats treated with CdCl_2_ had significantly lower serum SOD levels, consistent with prior studies by Nna *et al*. [10] and Dailiah and Padmalatha [56]. However, it has been demonstrated that PRO has potent antioxidant properties which lowers membrane phospholipid oxidation and peroxidation. The membrane’s resistance to metal exposure can be increased by inducing antioxidant enzymes, which can also strengthen the membrane’s integrity [57]. In the present study, a noteworthy increase in SOD activity was noted in the groups that received PRO treatment. Furthermore, PRO’s phenolic and flavonoid composition supports its antioxidant properties and scavenging capabilities, preventing oxidative damage to lipids and other [57, 58]. Bu *et al*. [59] have mentioned that quercetin (QE) is a potent natural antioxidant originated from plants such as onions, nuts, berries, cauliflower, cabbage, and many other foods. According to Unsal *et al*. [60], QE exhibits antioxidant activity by scavenging free radicals and enhancing the activities of antioxidants such as catalase, glutathione peroxidase, and SOD [59, 61]. QE’s antioxidant and anti-apoptotic properties provide multi-mechanistic protection for the female reproductive system against Cd damage [10]. Vitamin E (tocopherol) possesses biological properties, including antioxidant properties, that protect cellular macromolecules, such as DNA, proteins, and lipids, from free radicals [62]. Duan *et al*. [63] have demonstrated that the antioxidative and antiapoptotic properties of Vitamin E and metallothionein reduce the hepatotoxicity of Cd. PRO and Vitamin E act together to reduce the time-related damage that metals cause to the reproductive system [19].

### Histopathological restoration of the reproductive axis

In this study, the hypothalamic-pituitary-ovarian-uterine axis did not exhibit any obvious macroscopic pathological lesions. However, exposure to Cd induced notable histological alterations in the pituitary gland, including disrupted cell cord arrangement, edema, and hyperemia [33]. Cd exposure reduced ovarian weight. On the other hand, histological analysis of the ovaries of rats exposed to Cd without a PRO supplement revealed degenerative alterations in endocrine cells, with a decrease in interstitial cells and thickness in OSE and an increase in atretic follicles. This phenomenon may be caused by disruption of the hypothalamic-pituitary-ovarian axis caused by Cd toxicity, which, in turn, leads to a decrease in gonadotropin hormones (FSH and LH), which control histological and structural changes in the ovaries, including follicle numbers, weight, and diameter [64]. FSH and LH stimulate the production of ovarian growth factors and cytokines that prevent antral follicle apoptosis [65, 66].

A previous study by Ighodaro and Akinloye [52] demonstrated that Cd is implicated in impairing folliculogenesis in mammals, leading to a reduction in both the quantity and quality of ovulated oocytes, as well as hindering fertilization success. The primary mechanism by which Cd has detrimental effects on the development of ovarian follicles is by damaging granulosa cells, which are somatic cells that comprise ovarian follicles and play a crucial role in regulating follicle development [53, 67, 68]. Prior research on granulosa cells has demonstrated that Cd decreases the levels of gonadotropins, which act as an apoptotic inhibitor factor [69]. Therefore, Cd-induced cytotoxicity results in granulosa cell loss, leading to a decline in 17β-estradiol synthesis and hypoestrogenism [70]. A previous study by Kim *et al*. [71] revealed that the EDC Cd influences the hypothalamic-pituitary-ovarian axis, which alters hormone synthesis associated with polycystic ovary syndrome (PCOS). There is a relationship between blood Cd concentration and low levels of FSH [72]. Stimulatingly, PRO has been found to be a helpful treatment for restoring the ovarian structure and follicle development in rats with PCOS [73].

According to the present study, PRO supplementation significantly improved ovarian weight and conferred histological protection, as evidenced by a reduction in atretic follicles and preservation of the endocrine architecture. This effect has been attributed to the renewing and regulating characteristics of PRO [22, 74]. The study findings showed that a PRO supplement at doses ranging from 150 to 500 mg/kg BW was effective in boosting the growth and maturity of ovarian follicles as well as in raising the number of surviving follicles. The observed effects may be attributed to the diverse bioactive constituents of PRO, including polyphenols, terpenoids, flavonoids, and phe-nolic acids [18].

### OSE remodeling and endocrine cell regeneration

The current data indicate that the influence of Cd, which generates ROS, decreases the number of interstitial cells. A marked increase in the number of ovarian interstitial cells was noted in the PRO-treated groups, indicating enhanced cellular regeneration, indicating significant protection. This finding is consistent with a prior study that demonstrated that PRO extract promotes tissue regeneration and cell proliferation *in vitro* [75]. According to Nasiadek *et al*. [34], administering Cd to rats can also alter the levels of reproductive hormones like E2 and result in degenerative alterations in the endocrine cells of the Cd-treated group. This is consistent with the findings of the present study. Interestingly, the results of this study support those of Okamoto *et al*. [38], who found that oral PRO administration causes estrogenic activity in estrogen target organs *in vivo*. This suggests that PRO is a safe phytoestrogen supplement and a potential treatment for Cd toxicity. A previous study by revealed that triterpenoids have estrogenic properties, and derivatives of caffeic acid were found in PRO. In addition, PRO possesses estrogen-like properties *in vivo* [76].

Alterations in the hypothalamic–pituitary–gonadal axis affect steroidogenesis in the ovary’s interstitial and theca cells [77]. According to the present study, PRO appears to facilitate the upregulation of reproductive hormone synthesis, possibly through ER-mediated signaling pathways, indicating that PRO has a stronger effect on rat ovaries. A dose-dependent increase in serum E2 levels suggests that PRO has a beneficial impact on the synthesis of steroid hormones. However, the ovarian, uterus, and mammary glands have also been identified by Guzeloglu-Kayisli *et al*. [78] as steroid target organs. According to the present study, PRO increased the number of interstitial cells that generated androgen.

### Morphological remodeling of OSE

The impact of PRO supplementation on OSE subjected to Cd toxicity was also examined in this study. With the increase in the PRO supplement dose (between 150 and 500 mg/kg BW of PRO), OSE morphology transitioned from simple squamous to simple columnar epithelium with increasing PRO doses, indicating epithelial remodeling. The results also suggest that PRO may change the form of OSE affected by Cd poisoning. According to a prior study by Albishtue *et al*. [30], which used scientific evidence for edible bird’s nest (EBN)’s role in preserving the integrity of the histological structure of rat ovaries exposed to lead acetate, similar OSE morphological changes from simple squamous to columnar epithelium were reported in lead acetate-exposed rats supplemented with EBN [30]. EBN has been widely used by humans as a tonic and medicinal diet in traditional Chinese medicine. The nutritious components of EBN possess proteins, mineral salts, vitamins, hormones, and fatty acids [79]. EBN was used as a prophylactic hormonal replacement agent. Due to its epidermal growth factor (EGF)-like activity, EBN has a stimulating effect on cell development and regeneration [80].

According to research using electron microscopy and histochemistry, OSE contributes significantly to follicular rupture due to its lysosome-like inclusions, which generate proteolytic enzymes [81]. OSE cells possess high secretory potential and express numerous receptors involved in cellular proliferation and differentiation [82]. OSE cells have been shown to be responsive to insulin-like growth factor-1 (IGF-1). Consistent with the current study’s findings, prior research has shown that Cd-induced lowering of serum IGF-1 levels results in decreased thickness of the OSE [83]. Likewise, IGF-1 activity may be a factor in the ovarian epithelium’s increasing thickness [60, 84]. This growth factor is primarily associated with the synthesis of sex hormones [85]. This finding is consistent with an earlier study that demonstrated thicker OSE and higher growth factor expression in ovarian epithelium and stromal cells, which is associated with elevated E2 concentrations [30]. These provide other evidence to the effect of PRO on reproduction and fertility.

### Uterine protection and estrogenic influence of PRO

In the present study, 4 weeks of exposure to CdCl_2_ without a PRO supplement had a deleterious effect on UBWR. In addition, histopathological analysis of the uteri of rats exposed to Cd revealed necrosis, a decrease in the number of uterine glands, and destruction of luminal and glandular epithelia, which is in agreement with Quddus *et al*. [11]. The present study focused on the effects of short-term exposure to Cd toxicity on the uterus as well as the advantages of taking a PRO supplement to reduce the toxic effects of Cd. Interestingly, the results of this study support those of Okamoto *et al*. [38], who found that oral PRO administration significantly increased the weight of the uterus and uterine structures, including the thickness of the LE, in ovariectomized rats compared with the control group [38]. The uterine glands and cells of rats were more active after receiving PRO treatment. Furthermore, PRO has estrogenic properties and promotes the development of uterine glands. Further research has found that PRO enhances weight, immunological function, growth performance, and antioxidant status. PRO also has other essential properties, including anti-bacterial and anti-inflammatory properties [86, 87]. PRO contributes to reproductive system homeostasis by modulating inflammatory signaling pathways implicated in vascular dysfunction [88]. Detailed OSE morphological transitions and dose-dependent restoration of the uterine glandular structure are under-reported and novel findings.

## CONCLUSION

This study provides compelling evidence that PRO exerts significant protective effects against Cd-induced toxicity in the female reproductive system of rats. Key findings demonstrated that PRO supplementation effectively normalized estrous cycle irregularities, restored E2 and SOD levels, and preserved the histo-logical integrity of the ovarian, uterine, and pituitary tissues. Rats exposed to Cd showed prolonged estr-ous cycles, reduced ovarian and uterine weight rat-ios, increased atretic follicles, disrupted epithelial morphology, and elevated caspase-3 expression. These alterations were reversed in a dose-dependent manner by PRO, particularly at 500 mg/kg BW, which significantly increased follicular survival, VEGF expression, interstitial cell count, and epithelial remodeling in both the ovary and uterus.

The practical implications of these findings underscore the therapeutic potential of PRO as a natural, phytoestrogenic compound capable of mitigating heavy metal-induced reproductive dysfunction. Its ER modulatory activity, antioxidative capacity, and anti-apoptotic properties position PRO as a promising candidate for integrative strategies aimed at preserving reproductive health in populations exposed to environmental toxins.

A major strength of the study lies in its comprehensive evaluation of the hypothalamic–pituitary–ovarian–uterine axis using multiple parameters, including histological, biochemical, hormo-nal, and morphometric endpoints. The integration of both molecular and functional markers provides a mechanistic understanding of PRO’s protective action.

However, the study is limited by its short-term exposure design, use of a single animal model, and lack of molecular assays (e.g., Western blotting, gene expression) to quantify specific signaling pathways. In addition, the bioavailability and tissue distribution of PRO’s active constituents were not assessed.

Future research should aim to validate these findings in long-term and multi-species studies, eluc-idate molecular signaling cascades involved in PRO-mediated protection, and explore its translational potential through pharmacokinetic and toxicological evaluations in clinical or field-based settings.

This study reinforces the role of PRO as a natural modulator of reproductive toxicity and advocates its inclusion in nutraceutical or pharmaceutical interve-ntions aimed at mitigating Cd-induced reproductive impairments.

## DATA AVAILABILITY

All the generated data are included in the manuscript.

## AUTHORS’ CONTRIBUTIONS

AAA, AMA, HAA, and MAA: Conceptualized the study. AAA, AMA, and HAA: Conducted the study. AAA and AMA: Statistical analysis. AAA: Wrote the original manuscript. HAA, MAA, and AAA: Edited and reviewed the manuscript. All authors have read and approved the final manuscript.
